# An innovative approach for provenancing ancient white marbles: the contribution of x-ray diffraction to disentangling the origins of Göktepe and Carrara marbles

**DOI:** 10.1038/s41598-021-01800-7

**Published:** 2021-11-16

**Authors:** F. Antonelli, F. Nestola

**Affiliations:** 1 LAMA - Laboratory for Analysing Materials of Ancient Origin, IUAV University of Venice, San Polo, 2468, 30125 Venezia, Italy; 2 Department of Geosciences, University of Padova, Via G. Gradenigo 6, 35131 Padua, Italy

**Keywords:** Geochemistry, Mineralogy, Petrology

## Abstract

The paper presents a very efficient, quick, low-cost and minimally micro-destructive approach to discriminating between Roman artefacts sculpted with Göktepe (Aphrodisia, Turkey) or Carrara (Apuan Alps, Italy) white marbles by using a standard X-Ray Powder Diffractometer (XRPD) and a refinement of the unit cell parameters and volume of calcite. At present, the routine way of differentiating between these two almost indistinguishable by-eye marbles is based on the typically higher strontium content of calcite in the Microasiatic lithotype, a unique geochemical-crystallographic feature with respect to all other non-Göktepe fine-grained white marbles used in classical times. The XRPD approach has been verified by testing eighteen samples of known composition, nine from Carrara and nine from Göktepe quarries, which had already been analysed with other laboratory techniques. The applicability of the method to archaeological artefacts was confirmed by an archaeometric study performed on some famous Roman sculptures of the National Archaeological Museum of Venice and from Hadrian’s Villa at Tivoli. The results show that Göktepe/Carrara discrimination is always possible and that this XRPD approach can potentially become a useful and low-cost routine procedure to solve provenance issues.

## Introduction

The worldwide success of marble is inscribed in the intrinsic beauty of the material, which can be enhanced through different working processes, capable of generating numerous aesthetic results from the same type of marble. As is well known, the history of the use of crystalline white marble goes back thousands of years, starting already in prehistoric times. In particular, the creation of the first marble objects dates back to the late Neolithic, in the Cyclades (especially the islands of Paros and Naxos), where characteristic small idols/figurines, peculiar to the artistic production of the Cycladic civilization, were produced from the end of the IV to the end of the III millennium BC. White marbles outcrop copiously in various regions of the Mediterranean basin (Greece, Asia Minor, Italy, etc.), and their use was consequently widespread from the origins of archaic Greek sculpture and then, from the V century BC, in the architecture of the classical and later periods, when a huge amount of marble was employed and the quality of the craftsmanship reached extraordinary artistic levels. For the Roman elite, monumental marble sculpture and architecture offered potent emblematic and symbolic propaganda ways of exhibiting, upholding and amplifying their social status.

Archaeologists and art historians have always been extremely interested in the identification of the quarry of provenance of ancient marble artefacts, which is one the most hotly debated issues of petroarchaeometry. The devising of a reliable method for determining the provenance of marble has meant that sculptures of uncertain ascription can be attributed to a specific production area, workshop or artist (often offering valuable elements for proving their authenticity); marbles recovered from shipwrecked cargoes enable ancient trade routes to be reconstructed^1, 2^; buildings, statues, artefacts and monuments in general, if accurately dated, can in turn reveal when a specific quarry started its activity and how the demand for its marble developed; damaged architectural or sculptural marble elements can be restored, replaced or integrated with sound material sourced directly from the ancient quarries^2^.

For all these reasons, in the absence of an effective non-destructive technique, geologists (mainly petrologists and mineralogists) and scholars of other disciplines, especially chemists and physicists, have been trying for more than a century to achieve a consistent identification of the source areas by means of a unique or multiple laboratory analysis. To date, when marbles are found as original structural or decorative elements of ancient buildings or in sculptures, or when ancient spolia are reused in Medieval or Renaissance monuments (as in the cases of historical architecture of Rome, Venice and Istanbul), a multi-method approach offers the best means of successful identification, combining at least two different analytical methodologies and jointly processing all the data obtained^2^. In particular, the detailed mineralogical-petrographic examination of a thin section and the determination of the C and O stable isotopic ratios on the same sample is currently the most widely used and reliable combination. Such a combination exploits the most recently updated databases for the main Mediterranean marbles most commonly used in classical antiquity and it associates the global reference isotopic diagrams to the maximum grain size (MGS) of the different varieties^2^. When petrographic and isotopic features are compared step by step in this way it often proves possible to identify where the marble constituent of a given archaeological/historical object was originally quarried. We use the term “often” advisedly; it does not mean “always”.

### The discovery of Göktepe white marble and related issues

**Figure 1:**
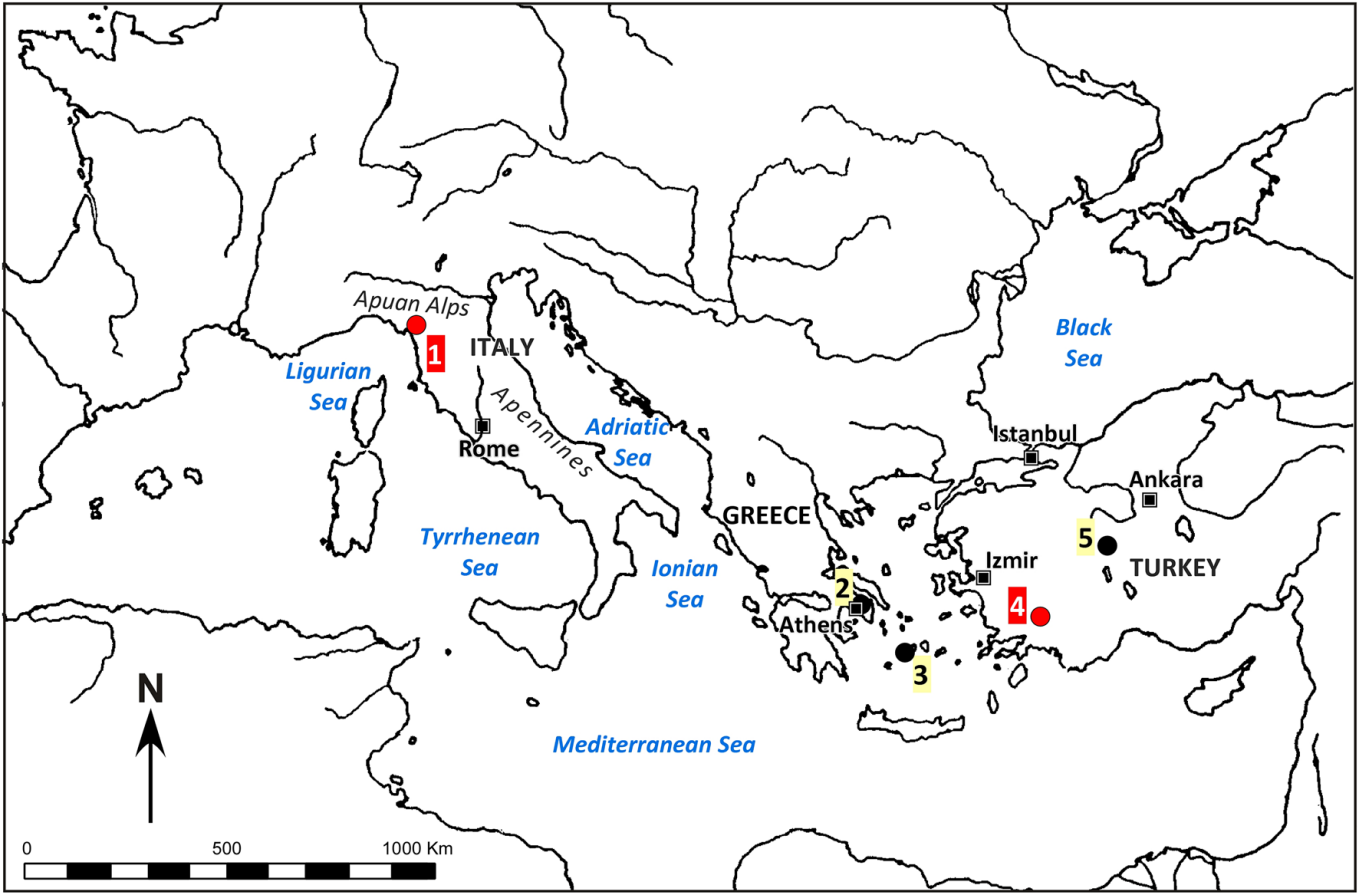
Map of the central to eastern Mediterranean region showing the location of the quarry sites of the most important fine-grained marbles used in classical times. 1. Carrara (Italy); 2. Mt. Penteli (Attica, Greece); 3. Paros island (Stephani; Greece); 4. Göktepe (Muğla, Turkey); 5. Docimium (Afyon, Turkey).

The recent discovery of the ancient marble quarries of Göktepe, located in Caria, 40 km south-west of Aphrodisias (SW Anatolia, Turkey)^3^, has raised attention and much discussion. The importance of this fine-grained white marble has been claimed as extraordinarily high by the authors who first published on the quarries^4–7^. Their analyses and deductions, however, have prompted arguments among some scholars, e.g.,^8–12^. These disagreements have concerned mainly the real extent to which Göktepe white marble was preferred for high quality sculptural purposes and also the methodology adopted in determining the provenance of these artefacts. In fact, further petrographic investigations^12, 13^ have shown that although the Göktepe marbles can exhibit a unique set of features, their fabrics and grain size are more variable than initially suggested and several types of other important classical fine-grained white marbles (Fig. 1) can be confused with them. In particular, the closest counterpart is Carrara marble, a lithotype quarried since the Etruscan-late Hellenistic period^14^ and very widely exploited during the Roman period. More recently, during the Italian Renaissance, Baroque and Neoclassical periods, it was the raw material of choice for statuary and other artefacts by master sculptors such as Michelangelo, Bernini and Canova.

**Figure 2:**
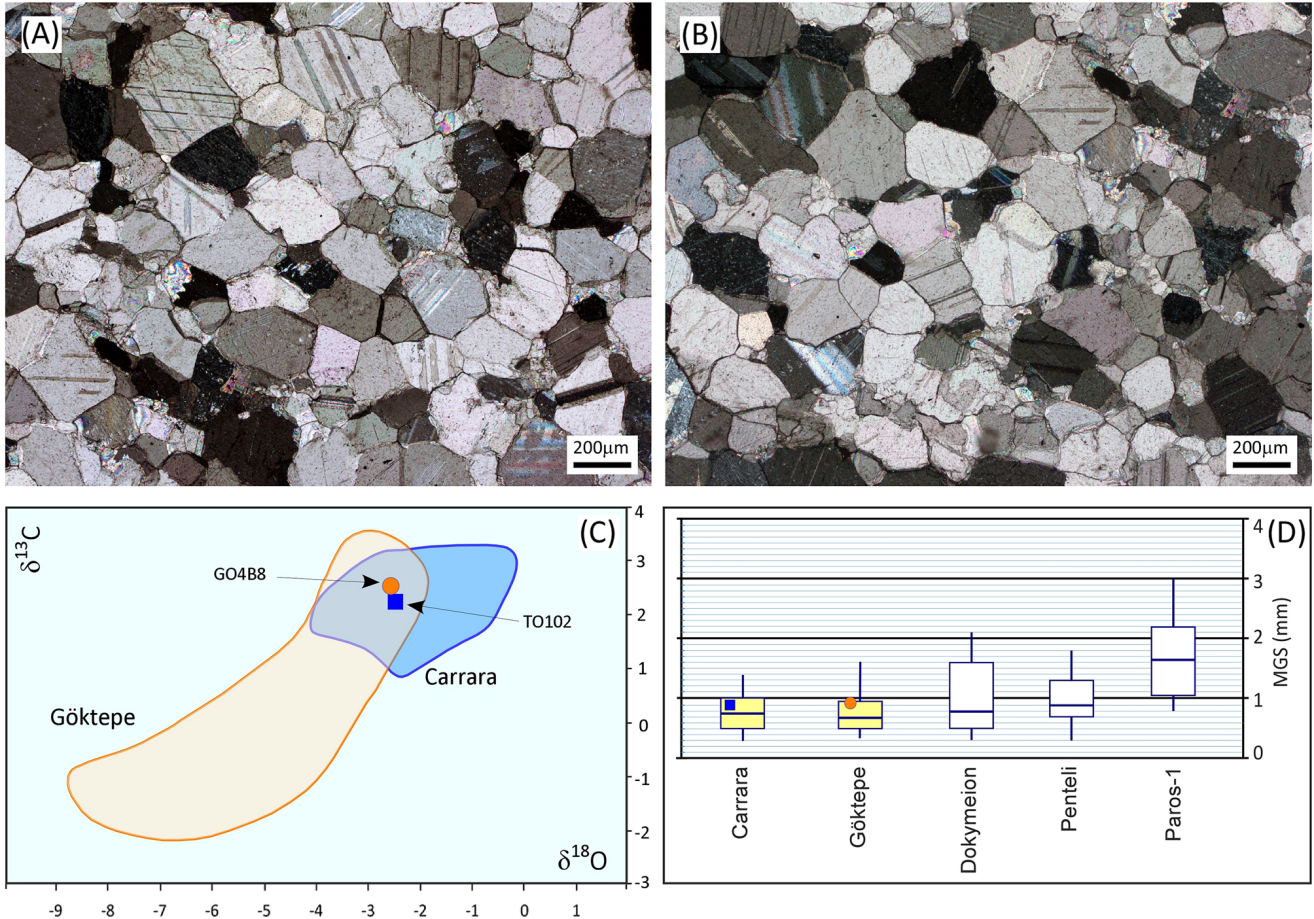
Comparison between the petrographic features shown by samples GO4B8 (**A**) and TO102 (B) from the Göktepe and Carrara quarries, respectively. (**A**,**B**), mosaics made of calcite crystals with straight to curved boundaries forming some triple points. (**C**), isotopic composition of the samples compared with the reference database (after Wielgosz-Rondolino et al.^12^, modified). (**D**), the reference maximum grain size (MGS) box bars of carbonate crystals for the five chief fine-grained white marbles used in antiquity (after Antonelli and Lazzarini^2^, modified). Boxes comprise 70% of the total values; the ends of the lower and upper whistlers represent the minimum and maximum values, respectively. The bar inside a box is the median value. The MGS of GO4B8 (circle) and TO102 (square) samples are also reported.

At first, the main parameters used to distinguish the Göktepe lithotype from other fine-grained white marbles were the variability of Sr and Mn concentrations, EPR (Electron Paramagnetic Resonance) measurements, and statistical elaboration of data. Geochemical studies performed on white marble from Göktepe, e.g.,^6, 12, 13, 15^ showed very high Sr and very low Mn concentrations, which were suggested as the key criteria for distinguishing Göktepe marble from other white fine-grained marbles used in antiquity, especially from *marmor lunense*, i.e. Carrara marble from the Apuan Alps in Italy. This is a critical point because the macroscopic characteristics, microscopic/petrographic features (fabric, microstructure, calcite boundary types, MGS/maximum grain size of the larger carbonate crystals) and O–C stable isotope composition (Fig. 2) of Carrara marble are often similar to those of Göktepe marble.

Nevertheless, despite the apparently wide range of analytical methods used for identification of Göktepe white marble, ambiguity still existed. For this reason, Wielgosz-Rondolino et al.^12^ proposed an improved methodology for the identification of Göktepe white marble and a consistent comparison with the Carrara type. The multi-method approach they proposed includes a detailed petrographic study in thin section, a cathodoluminoscopic characterization and a combination of isotope measurements with elemental ratios. The most important features of white Göktepe marble that arise from this study are: (a) a general homeoblastic fabric, sometimes with triple joints, often formed by small calcite crystals (MGS from 0.6 to 2.05 mm) mainly with curved/straight boundaries (all features which are shared with Carrara marble). Equally frequently, the fabric is heteroblastic and slightly foliated (frequently with traces of strain), at times showing very fine intergranular calcite grains and areas of decussate fabric related to rudist’ clast-relics. This set of petrographic characteristics together with the possible presence of small pink-to-light purple fluorite crystals, can be very peculiar; (b) oval domains and tiny spots with low intensity orange calcite in otherwise non-luminescent and dolomite-free marble; (c) tailed area on the stable C versus O isotope plot towards depleted values. Nevertheless, the isotopic domain partially overlaps with that of Carrara marble; (d) very narrow range of ^87^Sr/^86^Sr isotopic ratio around 0.7073; (e) low Mg, Mn, Fe and REE, but very high Sr contents.

As already suggested by Attanasio et al.^6^, Poretti et al.^15^ and Prochaska et al.^16^, the research conducted by Wielgosz-Rondolino et al.^12^ confirms that important geochemical parameters for discrimination between Carrara and Göktepe marbles are C and O isotope compositions, Sr and Mn contents, and Mg and trace element distribution, but it also indicates ^87^Sr/^86^Sr values as a further valuable proxy. Their data clearly show that the best discrimination between Göktepe, Carrara and other fine-grained white marbles is obtained when the Sr isotope signature is combined with Mn/Sr and Sr/Mg elemental ratios and δ^18^O values. Such combinations (proposed as diagrams by Wielgosz-Rondolino et al.^12^) can provide decisive results even when the individual proxies overlap, because when combined they should normally generate separate clouds of projection points.

## Aims and methods

So far, all the laboratory approaches proposed by different scholars to identify the Göktepe marble and, especially, to distinguish it from the Carrara variety, involve the joint use of several analytical techniques. These methods have some potentially major negative consequences: (1) the whole instrumental apparatus needed is quite composite and only a very small number of laboratories are adequately equipped; furthermore, the complete analytical procedure is not so effortless and quick as to be considered as a real “routine method”; (2) the multi-analytical method requires a not inconsiderable amount of sample material; in fact, Wielgosz-Rondolino et al.^12^ suggest that in the case of precious artefacts, when the petrographic investigations in thin section are not possible and least destructive methods are necessary, a combination of only Sr, C and O isotope analyses may be preferred, as they require less material than the elemental analysis performed by ICP spectrometry or atomic absorption; (3) the whole set of discriminant analyses entails a major cost per sample (many hundreds of euros).

This paper aims to describe a quick, low-cost and only minimally micro-destructive approach (potentially a routine procedure) able to distinguish between Göktepe and Carrara marbles on the basis of their dissimilar basic mineralogy (related to the different geochemical features) as revealed by the X-ray powder diffraction (XRPD) technique. A representative number of the powdered samples already used by Wielgosz-Rondolino et al.^12^ are reconsidered for this purpose. In particular, in order to provide robust statistical data, we have analysed 18 different samples, 9 marbles from the district of the Carrara quarries (samples from Bacchiotto, Crestola, Calagio, Gioia, Ravaccione, Torano, and Fantiscritti) and 9 marbles from the Göktepe area (samples from quarries 2D, 3, and 4^12^).

XRPD measurements were performed using a Philips X’Pert Pro MPD diffractometer. This instrument implements a long fine focus Co anode working at 40 kV–40 mA and a goniometer with radius of 240 mm that operates in the θ/θ geometry. Incident beam optics include the Bragg-BrentanoHD (BBHD) module that is a wafer crystal of W/Si, manufactured to improve signal-to-noise and peak-to-background ratios. It is also designed to maintain a divergent beam, reducing K_α2_, to suppress K_β_ lines and thus avoiding the use of an Fe filter. Divergence slits of 0.25°, antiscatter slits of 1° and Soller slits of 0.04 rad complete the incident beam setup.

Six samples were split and XRPD analyses replicated also by means of a PANalytical Empyeran multi-function diffractometer, which implements a Copper anode. The "PANalytical Empyeran" diffractometer worked under the same operating condition used for the "Philips X’Pert Pro MPD"; the results obtained were perfectly in agreement and confirm that there was no instrumental bias.

Diffracted beam optics are composed of an antiscatter slit of 9.1 mm aperture, Soller slits of 0.04 rad and X’Celerator Position Sensitive Detector with a 2.122° 2θ active length. Total scan time per sample is approximatively 2.5 h and 1 h when using "Philips X’Pert Pro MPD" and PANalytical Empyeran multi-function diffractometers, respectively. A beam knife, positioned above the sample centreline, was used to reduce the air scattering contribution at low 2θ angle.

Measures were carried out at angles of between 3° and 95° 2θ, using a 0.008° step size, counting 100 s per virtual step on a spinning sample (1 revolution per second). Samples were prepared using the back-loading procedure in order to reduce crystallite preferred orientations.

The fundamental parameters approach^17^ as implemented in Profex-BGMN v.4.1.0^18^ was used in the Rietveld refinements. A preliminary verification step consisted in checking the instrumental contribution to line broadening by fitting the diffraction profile of the Si standard (NIST SRM-640c). This procedure involved the verification of FPA configuration bundled with software, after considering the presence of the BBHD module. Crystal structures were taken from the BGMN structure database. The refinement strategy included the variations of the following parameters: coefficients of the Lagrangian polynomial function used to describe the background (6 coefficients for each sample), scale factors, unit cell parameters, crystallite size, texture and microstrain. Neither atomic coordinates nor atomic displacement parameters were refined.

## Results and discussion

**Figure 3:**
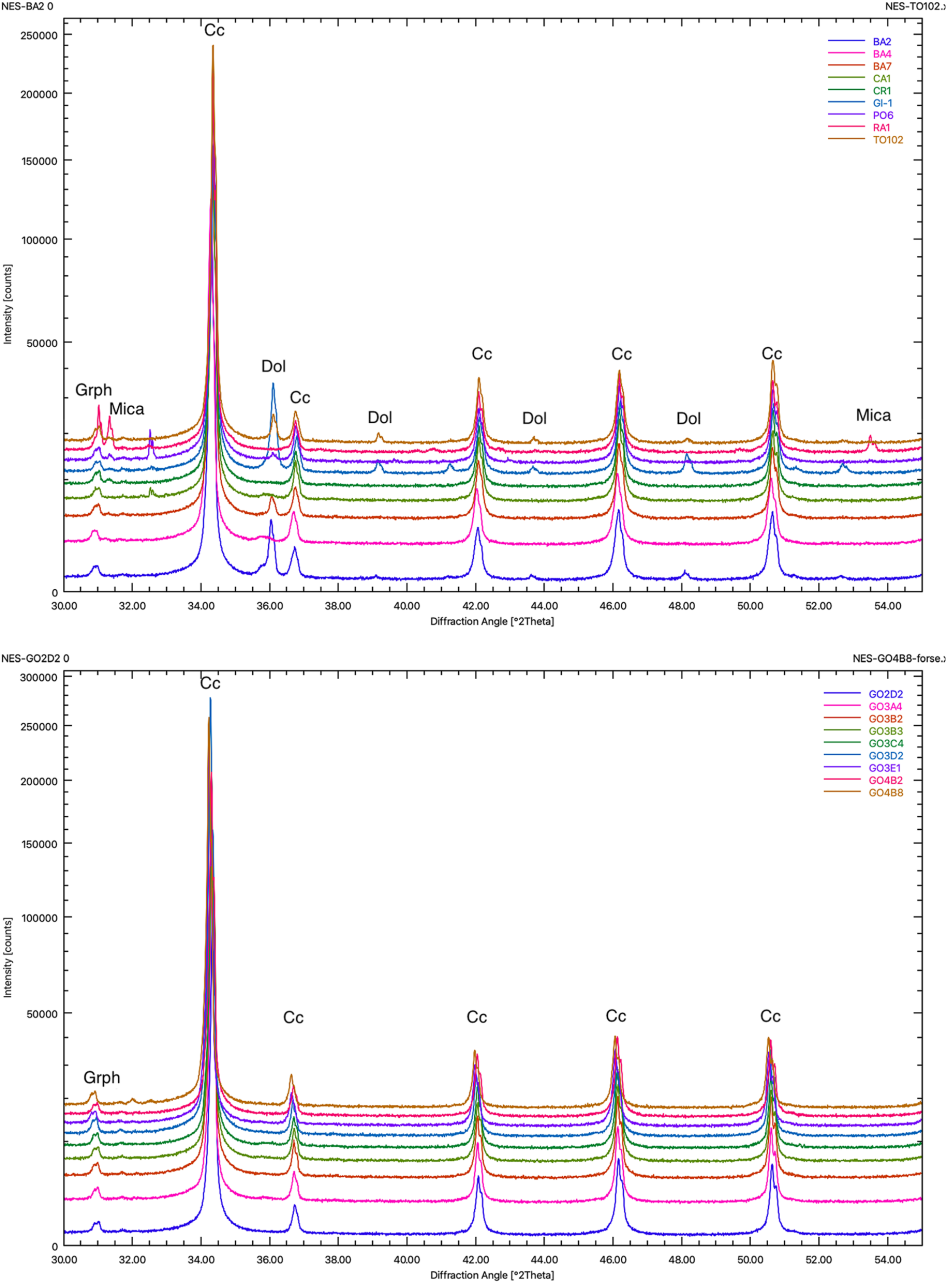
X-ray powder diffractograms of 18 marble samples from Carrara (image on the top) and Göktepe (image on the bottom). Symbols: Cc = calcite; Grph = graphite; Dol = dolomite; a few diffraction peaks can be assigned to a mica. The small peaks at about 32.6° can be assigned to plagioclase, which could be present in traces.

**Figure 4:**
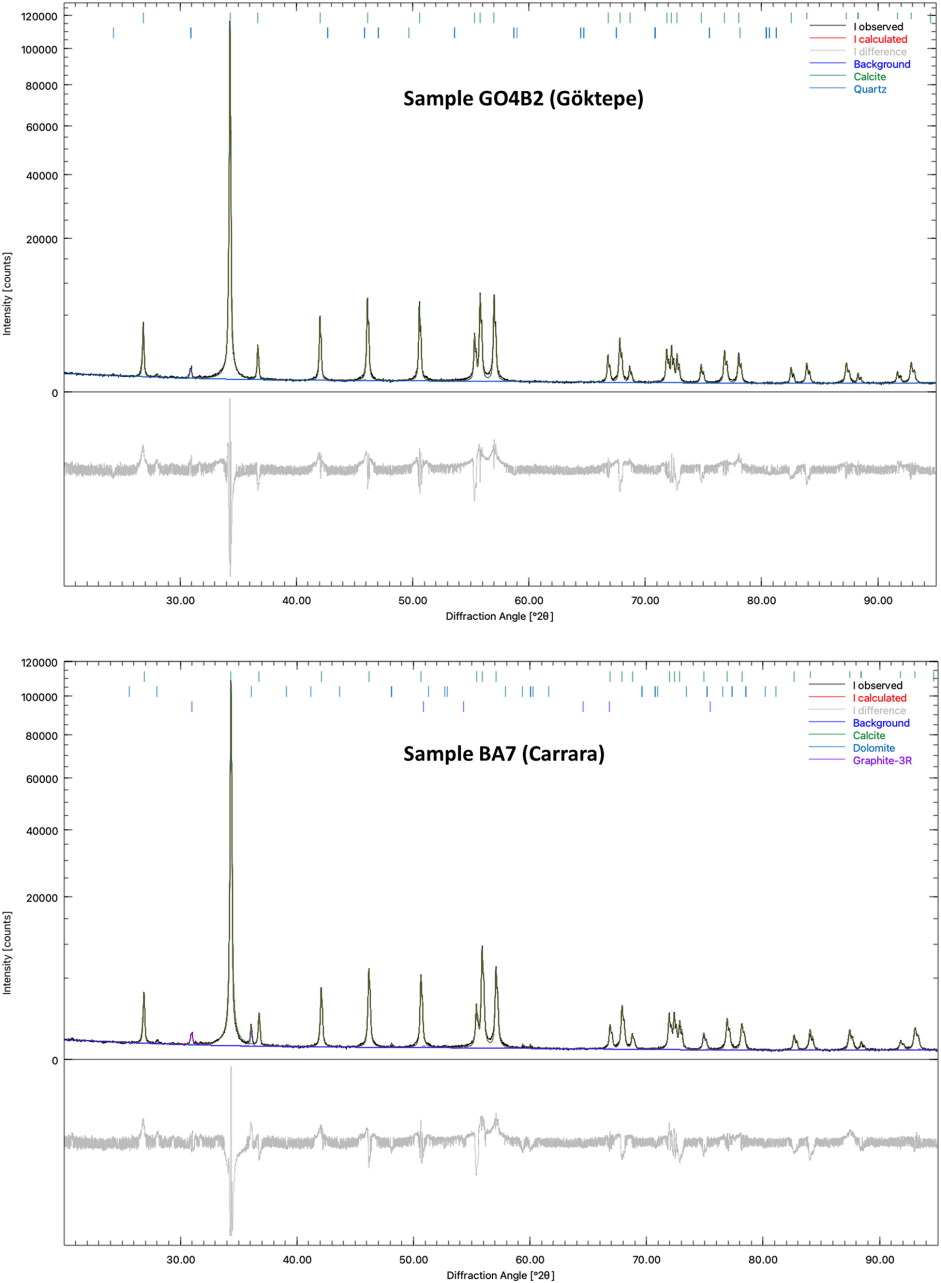
Refined diffractograms and their residuals plot for sample BA7 of Carrara and GO4B2 of Göktepe.

X-ray powder diffractograms relative to all 18 samples are shown in Fig. 3. Two refined diffractograms and their residuals plots are shown in Fig. 4. The data show conclusively that marble from Göktepe is nearly pure calcite with a negligible amount of graphite as impurity; on the other hand, marble from Carrara is definitely a more impure marble, often with a significant presence of dolomite, graphite, mica and traces of plagioclase and quartz. Figure 3 certainly provides a clear and easy way of distinguishing the two types of marbles, based only on sample purity.

In general, some varieties of Carrara marble that are extremely pure can certainly exist; consequently, this first very simple XRPD approach would probably not be sufficient to avoid potential identification problems. However, a detailed analysis of the calcite structure in terms of unit cell parameters could provide more information. As we remarked in the introduction, marbles from Göktepe generally show much higher Sr contents; Sr is a larger cation than Ca (e.g., calcite crystal structure has Ca in 6-coordination and it is well established that six-coordinated Sr has a cation radius equal to 1.18 Å against 1.00 Å of Ca, see Shannon^19^) and this should cause a slight but detectable increase in the unit-cell parameters and volume of calcite. In order to verify such possible influence on the volume (and on the *a* and *c* cell parameters), we have refined the unit-cell parameters of all 18 samples and the relative data are reported in Table 1.

**Table 1:** Refined values for *a* and *c* cell parameters and unit-cell volumes of all 18 quarry samples and six archaeological artefacts.

Sample	a (Å)	EDS (Å)	c (Å)	EDS (Å)	V (Å)	EDS (Å)
**Carrara**						
BA2	4.98055	0.00008	17.01496	0.00029	365.523	0.002
BA4	4.97912	0.00005	17.00987	0.00019	365.204	0.002
BA7	4.98183	0.00004	17.02235	0.00014	365.871	0.001
CA1	4.98363	0.00004	17.03007	0.00014	366.301	0.001
CR1	4.98225	0.00004	17.02427	0.00013	365.974	0.001
GI-1	4.98021	0.00004	17.01394	0.00015	365.453	0.001
PO6	4.98070	0.00004	17.01797	0.00014	365.612	0.001
RA1	4.98235	0.00003	17.02542	0.00012	366.013	0.001
TO102	4.98274	0.00004	17.02903	0.00016	366.149	0.001
**Göktepe**						
GO2D2	4.98593	0.00003	17.04260	0.00012	366.909	0.001
GO3A4	4.98626	0.00003	17.04383	0.00011	366.984	0.001
GO3B2	4.98681	0.00004	17.04639	0.00013	367.121	0.001
GO3B3	4.98676	0.00003	17.04586	0.00011	367.101	0.001
GO3C4	4.98728	0.00003	17.04905	0.00011	367.247	0.001
GO3D2	4.98635	0.00004	17.04451	0.00013	367.012	0.001
GO3E1	4.98704	0.00003	17.04722	0.00012	367.172	0.001
GO4B2	4.98739	0.00003	17.04914	0.00011	367.265	0.001
GO4B8	4.98755	0.00003	17.04913	0.00011	367.289	0.001
**Archaeological artefacts**						
Dead Galatian	4.98825	0.00004	17.05372	0.00020	367.490	0.001
Kneeling Galatian	4.98888	0.00005	17.05757	0.00034	367.667	0.002
Falling Galatian	4.98188	0.00005	17.02372	0.00023	365.908	0.002
TI-VA19	4.98873	0.00001	17.05027	0.00039	367.487	0.002
TI-VA34	4.98229	0.00007	17.02516	0.00025	365.999	0.002
TI-VA62	4.98767	0.00009	17.04913	0.00036	367.306	0.003

*EDS* extreme studentized deviate.

**Figure 5:**
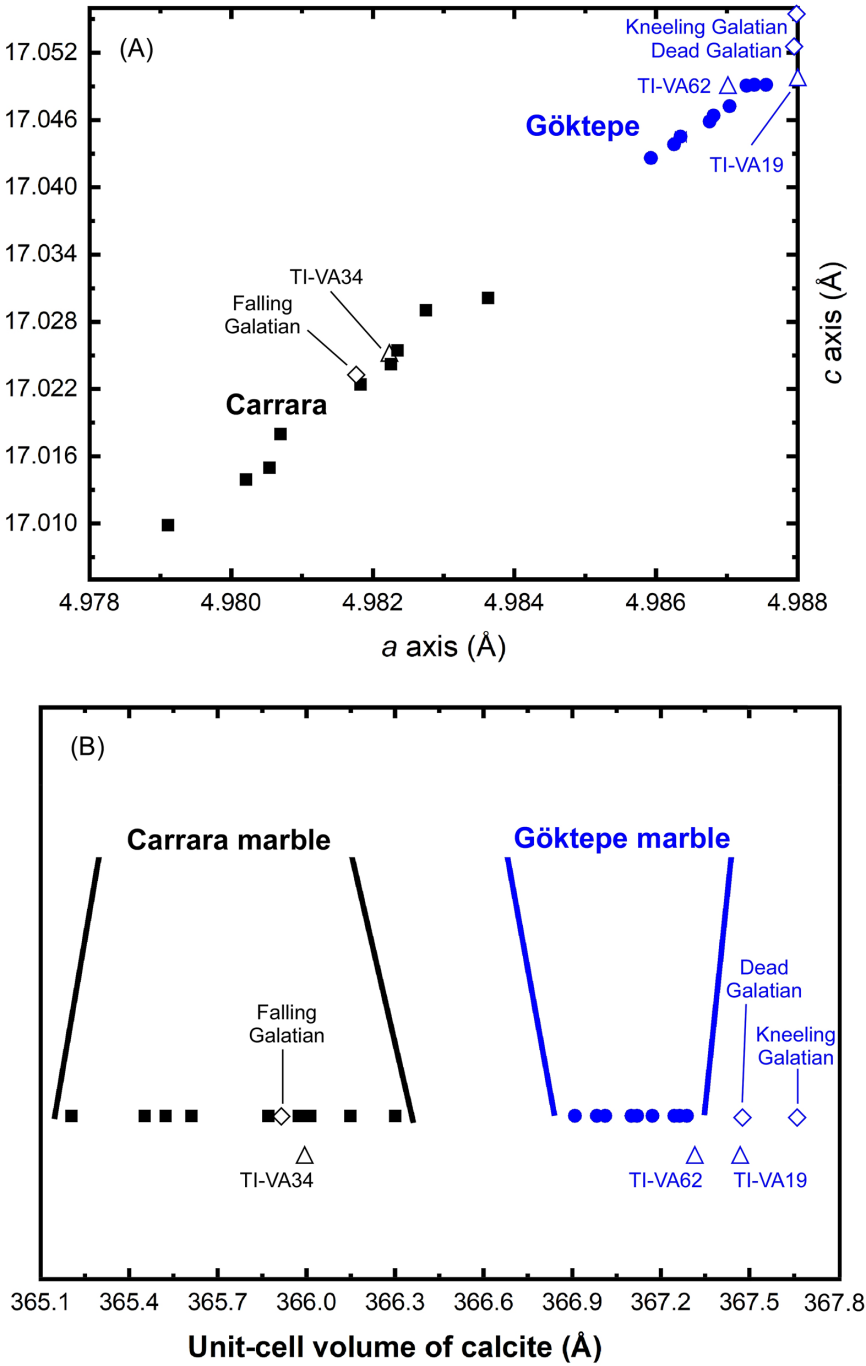
Plotting of refined data for *a* versus *c* axes (**A**) and refined unit-cell volumes (**B**) of the calcite unit-cells for the 18 marble quarry samples and six archaeological artefacts investigated here. Samples of Carrara and Göktepe marbles are in black squares and in blue circles, respectively; statues of Galatian soldiers and sculptures from Hadrian’s Villa are in diamonds and triangles, respectively.

The unit-cell parameters show conclusively that both *a* and *c* axes are slightly but significantly larger for Göktepe samples as can also be clearly seen in Fig. 5A. As a consequence of the behaviour of *a* and *c*, we also observe a larger unit-cell volume (Table 1 and Fig. 5B). Figure 5B shows a clear separation between Göktepe (in blue) and Carrara (in black) samples. Both Fig. 5A,B also show that calcite in Carrara marble displays lower *a* and *c* cell parameters and a higher volume variation within the refined values. It should be noted that this volume variation could be related to a higher chemical heterogeneity of calcite in Carrara marble with respect to that of Göktepe. To be remarked that even the largest volume among the samples from Carrara, i.e. sample CA1 in Table 1, does not reach the smallest volume among the samples from Göktepe, i.e. sample GO2D2 in Table 1. Considering the experimental uncertainties of the volume data reported in Table 1 (e.g., σV = 0.001 Å), samples CA1 and GO2D2 show differences in volume equal to 600 times their uncertainties. The largest differences in unit-cell volumes reach even 2000 times their uncertainties. This shows that the volume differences due to Sr content into the lattice are easily detectable. As a consequence of the volume behaviour, the *a* and *c* cell parameters also show differences between Carrara and Göktepe samples which are well beyond the experimental uncertainties and thus can be used as discriminating parameters.

Based on our results, we can state conclusively that the high Sr content within the calcite crystal structure from Göktepe samples increases the unit-cell volume slightly but significantly with respect to calcite in Carrara samples allowing a very easy identification between the two types of marbles regardless of all other minor minerals that could be present in the rocks from both the localities.

### Applicability of the method to archaeological artefacts

**Figure 6:**
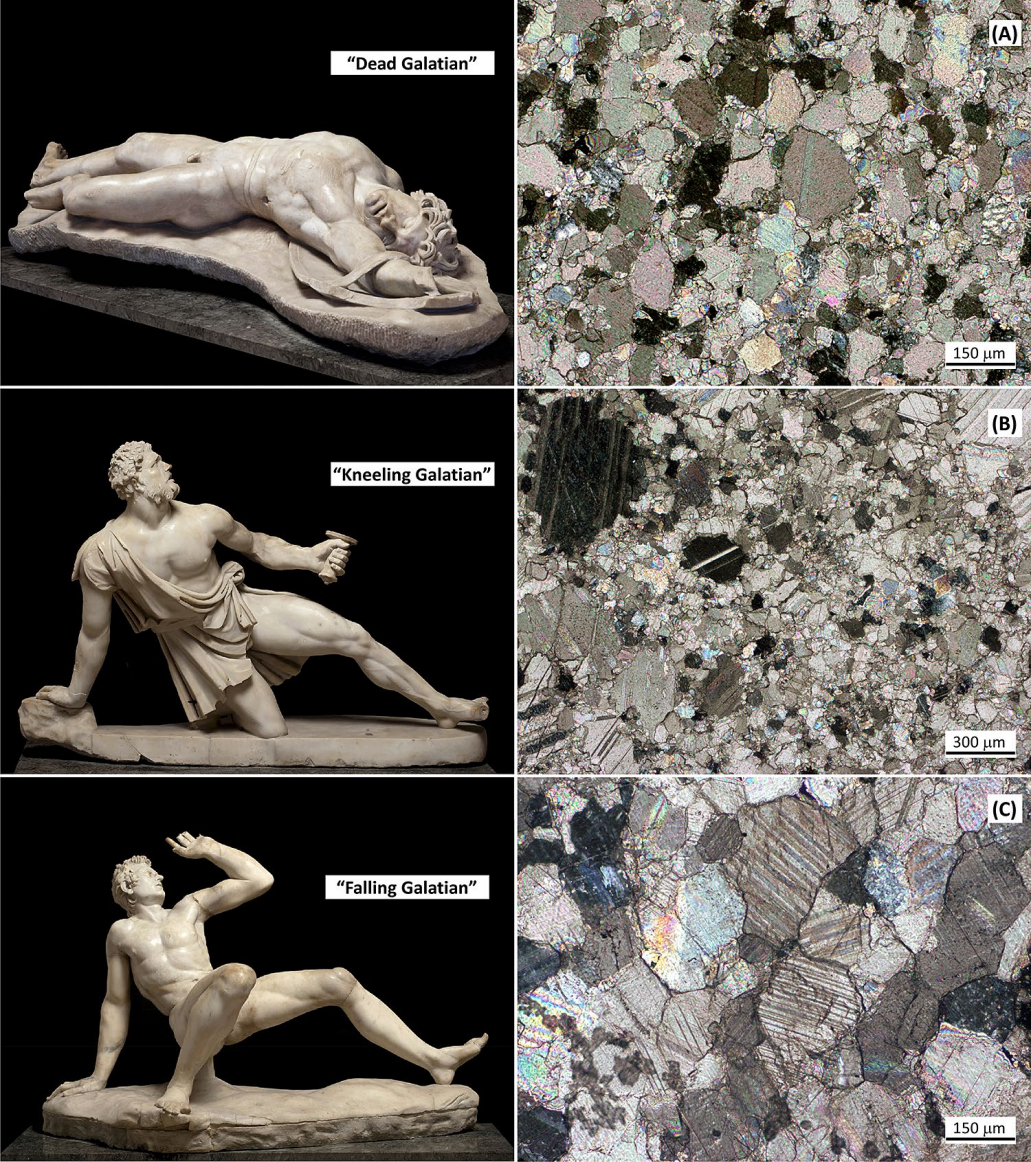
The three under-life-size sculptures of Galatian barbarians of the National Archaeological Museum of Venice and the petrographic features of their marble under the microscope. (**A**) homeoblastic mosaics made of calcite crystals with mainly curved boundaries; very fine-grained intergranular calcite grains are also visible. (**B**) as in figure (**A**) but with some coarse-grained calcite crystals tending to form a heteroblastic fabric. (**C**) homeoblastic mosaics made of carbonate crystals with straight and curved boundaries forming rare triple points.

The applicability of these proxies was tested on several archaeological artefacts for which Göktepe and Carrara provenance had already been proposed by different Scholars. We considered:

1. Three famous under-lifesize sculptures of Galatian soldiers (Gauls) defeated in combat, exhibited at the National Archaeological Museum of Venice (Fig. 6). According to Brunn^20^ and Overbeck^21^ it is generally agreed that ten Roman marble copies of the bronze statues of barbarians originally pertinent to a group of sculptures erected by a Pergamene king (Attalos I or Attalos II) on the Acropolis of Athens in Hellenistic times were initially present in Rome; they are now scattered in many cities and three of them probably correspond to those in Venice today^20^: the “Dead Galatian” (inv. No. 56), the “Kneeling Galatian” (inv. no. 57), and the “Falling Galatian” (inv. no. 55); the first two were previously analysed by Attanasio et al.^22^ and supposed to have been carved in Göktepe marble on the basis of isotopic and EPR data combined with Sr content (even if with some undesirable analytical anomalies in the case of the “Kneeling Galatian”), while the third one had never been studied before. Information on the discovery of these three “little barbarians” is not completely clear. We know that the “Falling Galatian” and the “Kneeling Galatian” were found by Cardinal Domenico Grimani in the early sixteenth century, perhaps on the northern side of the Quirinal Hill^23^ or around the Baths of Agrippa^24^, located in the Campus Martius, where Grimani had already discovered other antiquities. Toward the end of his life Domenico Grimani donated his collection to the Venetian state. By contrast, we have no exact information about the discovery of the “Dead Galatian”, even if generally it is assumed that this sculpture was found along with the other two.
2. Three samples of artefacts from Hadrian’s Villa in Tivoli (Ti-VA-19: base on legs; Ti-VA-62: *tondo* with zodiac; and Ti-VA-34: large mask) kindly provided by Maria Pilar Lapuente Mercadal (University of Zaragoza, Spain), who previously analysed them by means of petrographic, geochemical and physical (cathodoluminescence) techniques and hypothesised an origin from Göktepe for Ti-VA-19 and Ti-VA-62, and from Carrara in the case of sample Ti-VA-34,^13, 25^. The same geographic provenance of the marbles was confirmed successively by Wielgosz-Rondolino et al.^12^ also by using Sr isotopes.

As mentioned above, petrographic data concerning the marble artefacts from Hadrian’s Villa had already been collected. On the contrary, a thin section microscopic study of the marble used for the three Venetian sculptures of Galatian soldiers had not yet been carried out and it is presented here for the first time, as follows (Fig. 6).

The marble of the dead (inv. no. 56) and kneeling (inv. no. 57) Galatians is very fine-grained (grain-size mainly below 0.2 mm), with MGS values of 0.30 mm and 0.76 mm, respectively, and shows an homeoblastic mosaic made of calcite crystals with curved (± straight ± embayed) boundaries. Very fine intergranular calcite grains are also present (Figs. 6A,B); some coarser grain-size areas featuring a heteroblastic mosaic (Fig. 6B) formed by calcite crystals with abundant polysynthetic twinnings are also present in the marble of the “Kneeling Galatian”. In general, these petrographic features fit well with those described in literature for the Göktepe white marble. Conversely, a more regular homeoblastic fabric made of fine grains of calcite (and dolomite) with straight and curved boundaries, sometimes forming triple points, characterises the marble of the “Falling Galatian” statue (Fig. 6C); it shows an MGS of 0.60 mm and a more homogeneous and coarse grain-size with respect to the marble used for the other two sculptures of Galatians. These petrographic features are not dissimilar from those shown by the classical *marmor lunense*.

The results of the XRPD analyses carried out on the six archaeological artefacts showed that the marble used for the “Dead Galatian”, “Kneeling Galatian” and samples Ti-VA-19 and Ti-VA-62 is pure calcite and without impurities with respect to that of the Venetian “Falling Galatian” and sample Ti-VA-34 from Hadrian’s Villa, which also contains some amounts of dolomite and graphite. Furthermore, these four artefacts present similar refined calcite unit-cell parameters (Table 1), which match those recorded for Göktepe marble (Figs. 5A,B) very well; on the contrary, the “Falling Galatian” and sample Ti-VA-34 feature a smaller calcite unit-cell volume (as a consequence of the smaller dimensions of axes *a* and *c*), which are consistent with those established for Carrara marble (Figs. 5A,B). We can conclude that the “XRPD unit-cell refinement” method presented here fully confirms the assumed provenance of the marbles of the archaeological artefacts previously determined by Attanasio et al.^22^, Lapuente et al.^25^, Brilli et al.^13^ and Wielgosz-Rondolino et al^12^, by means of multi-analytical processes.

## Conclusions

The data we collected confirm conclusively that marble from Göktepe is nearly pure calcite (this was already known from literature data) as opposed to the marble from Carrara, which is much less pure. Though this first general mineralogical difference was very easy to observe by X-ray powder diffraction, account must be taken of the fact that, albeit very rarely, some varieties of Carrara marble can contain equally pure calcite; this can restrict the chance of safe discrimination between the two marbles by using only the qualitative XRD technique. However, the most significant result comes from the unit-cell refinements of calcite from the marbles of both localities. These are certainly new data that have never been reported before as a direct comparison between the two lithotypes.

We should remark that the unit-cell volume of a mineral is generally affected by the chemical composition of the mineral itself and, therefore, in the studied case, we can easily observe that the higher amount of Sr within the calcite structure of Göktepe marble with respect to that from Carrara systematically causes a larger cell volume (this can be expected as Sr is a much larger cation than Ca). Evidently, such approach is considerably more significant than a simple X-ray powder diffraction data collection because it is carried out only on calcite and is independent of the presence of further minerals in the rock. This means that, when a Göktepe versus Carrara (or non-Göktepe) origin is hypothesised, due to different chemical composition among different calcites, it will be possible to retrieve a reliable and distinguishable discriminant feature with a very limited amount of powder sample (starting from 5 mg), at much lower costs with respect to other laboratory techniques, lower measurement times, and very easy sample preparation (on average, for sample preparation, X-ray data collection and cell parameter refinement, we spent around 3 h per sample). Thus, only if this kind of simple XRD analysis indicates a non-Göktepe origin will it be necessary also to consider the classic thin section microscopic study supplemented by the Carbon and Oxygen isotopic signature (potentially carried out on the same powder used for XRD analysis).

The validity of the approach was confirmed by the results obtained on six archaeological samples already analysed throughout different and more elaborate chemical-physical laboratory techniques. Two of the “little Barbarians” statues of the National Archaeological Museum of Venice and two sculptures from Hadrian’s Villa in Tivoli were found to be made of Göktepe marble, whereas the remaining artefacts revealed unit-cell parameters which match those recorded for the Carrara marble. Furthermore, in the case of the so-called “Falling Galatian” sculpture of the Venetian National Archaeological Museum, our analyses support an origin of the marble which is certainly different from the Göktepe quarries and allows the use of *marmor lunense* to be hypothesized. These data show that the Venetian group of sculptures of “Galatian soldiers” is not homogeneous and, more in general, that Roman marble copies of the bronze statues of barbarians originally erected on the Acropolis of Athens were not produced only with Microasiatic marbles.

Further steps in this research will be (1) to extend the XRPD study of the calcite cell parameters/volume to the other fine-grained white marbles used in classical times—especially, the *Docimium* marble, i.e. the other important Microasiatic variety, but also the Greek Paros-1 (island of Paros, Cyclades) and Pentelic (Mt. Pentelicus, Attica) marbles—to verify possible differentiations and complete the database; (2) to apply and test this method on a more significant number of archaeological and museum artefacts for which an uncertain Göktepe origin has been proposed.
